# High Frequency of Lead Exposure in the Population of an Endangered Australian Top Predator, the Tasmanian Wedge‐Tailed Eagle (*Aquila audax fleayi*)

**DOI:** 10.1002/etc.4914

**Published:** 2020-12-03

**Authors:** James M. Pay, Todd E. Katzner, Clare E. Hawkins, Amelia J. Koch, Jason M. Wiersma, William E. Brown, Nick J. Mooney, Elissa Z. Cameron

**Affiliations:** ^1^ School of Natural Sciences University of Tasmania Hobart Tasmania Australia; ^2^ Forest and Rangeland Ecosystem Science Center, US Geological Survey Boise Idaho USA; ^3^ Forest Practices Authority Hobart Tasmania Australia; ^4^ Department of Primary Industries, Parks, Water and Environment Hobart Tasmania Australia; ^5^ Birdlife Australia Raptor Group, Birdlife Australia Carlton Victoria Australia; ^6^ School of Biological Sciences University of Canterbury Christchurch Canterbury New Zealand

**Keywords:** Avian toxicity, Environmental toxicology, Isotopes, Lead‐based ammunition, Wildlife toxicology

## Abstract

Lead poisoning, mainly through incidental ingestion of lead ammunition in carcasses, is a threat to scavenging and predatory bird species worldwide. In Australia, shooting for animal control is widespread, and a range of native scavenging species are susceptible to lead exposure. However, the prevalence of lead exposure in Australia's scavenging and predatory birds is largely unknown. We evaluated the degree to which the Tasmanian wedge‐tailed eagle (*Aquila audax fleayi*), an endangered Australian raptor and facultative scavenger, showed evidence of lead exposure. We detected lead in 100% of femur and liver tissues of 109 eagle carcasses opportunistically collected throughout Tasmania between 1996 and 2018. Concentrations were elevated in 10% of 106 liver (>6 mg/kg dry wt) and 4% of 108 femur (>10 mg/kg dry wt) samples. We also detected lead in 96% of blood samples taken from 24 live nestlings, with 8% at elevated concentrations (>10 μg/dL). Of the liver samples with elevated lead, 73% had lead^207/206^ isotope ratios within the published range of lead‐based bullets available in Tasmania. These first comprehensive data on lead exposure of an Australian raptor are comparable to those for raptor studies elsewhere that identify lead‐based ammunition exposure as a conservation threat. Our findings highlight the importance of further research and efforts to address lead contamination throughout the Tasmanian ecosystem and in other Australian regions. *Environ Toxicol Chem* 2021;40:219–230. © 2020 The Authors. *Environmental Toxicology and Chemistry* published by Wiley Periodicals LLC on behalf of SETAC. This article has been contributed to by US Government employees and their work is in the public domain in the USA.

## INTRODUCTION

Lead is a toxic metal that can negatively affect a range of physiological systems, thereby threatening susceptible animal populations and ecosystems (Goyer and Clarkson 2001; Finkelstein et al. 2012). Clinical signs of lead poisoning include ataxia, impaired mobility, lowered sensorial ability, vomiting, anemia, lethargy, gastrointestinal stasis, weakness, and mortality (Fallon et al. 2017). Although lead occurs naturally (Turekian and Wedepohl 1961), most bioavailable lead is brought into the environment through anthropogenic activities, such as mining, sewage treatment, paint, ammunition, and burning of fossil fuels (Schuhmacher et al. 1996; Udom et al. 2004; Finkelstein et al. 2012; Mackay et al. 2013). The worldwide distribution of this array of anthropogenic sources of lead has resulted in the documented lead exposure of >120 bird species (Haig et al. 2014).

One of the ways that avian predators and scavengers are susceptible to lead poisoning is through the incidental ingestion of lead‐based bullet fragments (Fisher et al. 2006; Golden et al. 2016; Pain et al. 2019). Although this has long been known, the use of lead‐based bullets remains prevalent worldwide, because the physical properties of this metal make it a suitable, inexpensive, and easy to manufacture projectile. When a lead‐based bullet hits a target animal, the bullet can fragment into small pieces that can be inadvertently ingested by scavengers (Hunt et al. 2006; Herring et al. 2016). Scavenging bird species are particularly prone to poisoning from these fragments, because they are often the first species to locate a carcass, the bullet wound provides an easy access point for feeding, and their highly acidic digestive tracts break down lead effectively (Beasley et al. 2015; Nadjafzadeh et al. 2015). Lead exposure has been reported in 34 raptor species (Pain et al. 2009, 2019), ingestion has been identified as the route of exposure (e.g., Katzner et al. 2017), and the link between lead‐based ammunition and exposure is evidenced by increased lead concentrations in raptors during the hunting season (Kelly and Johnson 2011; Cruz‐Martinez et al. 2012; Garbett et al. 2018), isotopic similarities between lead in tissues and those of ammunitions (Finkelstein et al. 2012; Ishii et al. 2017), and the presence of lead ammunition in regurgitated pellets and the alimentary canals of a range of raptor species (e.g., Donázar et al. 2002; Helander et al. 2009).

Lead can be detected in a range of tissue types, with each indicative of different types of exposure. Following lead fragment ingestion and absorption, lead is transported by the bloodstream and deposited primarily in liver, kidney, and bone (Pain et al. 2005). However, the persistence of lead in each tissue varies. The presumed relatively short persistence of lead in blood (≈13 d; Fry et al. 2009) and liver (days to months; Fisher et al. 2006) provides a measure of short‐term exposure, whereas bone lead is, to a large degree, an aggregate of lifetime exposure (although lead does travel in reverse from bone to blood; Fisher et al. 2006). These differences have been used to investigate acute and chronic exposures in raptor populations (Jenni et al. 2015; Behmke et al. 2017; Ganz et al. 2018).

The paucity of peer‐reviewed study of the rates and consequences of lead exposure of avian scavengers in Australia contrasts with increasing recognition of the issue in Europe, North and South America, Asia, and Africa. In Australia, a range of native scavengers are susceptible to exposure (Hampton et al. 2017), and the use of shooting for consumptive and nonconsumptive purposes is widespread. For example, estimates indicate that >1 million macropods (*Macropus rufogriseus* and *Thylogale billardierii*) and 400 000 brushtail possums (*Trichosurus vulpecula*) are shot annually across the Australian island state of Tasmania (Department of Primary Industries, Parks, Water and Environment 2011). Prior to the 1990s, the majority of these animals were shot for commercial harvest of skin and meat and were thus removed from the site after shooting (Department of Primary Industries, Parks, Water and Environment, Hobart, TAS, Australia, unpublished data). However, this harvest has since declined and now these animals are primarily shot to control population size to limit damage to agricultural and forestry assets (Department of Primary Industries, Parks, Water and Environment, Hobart, TAS, Australia, unpublished data). In these cases, the standard practice is to shoot the animal and to leave entire carcasses where they are shot, in situ. These factors, and similar behaviors nationwide, combine to potentially give Australia one of the most pervasive and abundant sources of ingestible lead material in the world (Hampton et al. 2017).

As a top avian predator and facultative scavenger, the wedge‐tailed eagle (*Aquila audax*) shares the same characteristics of avian scavengers on other continents that are threatened by lead exposure. Recent preliminary work has demonstrated that wedge‐tailed eagles on mainland Australia are exposed to lead (Lohr et al. 2020), and there is now growing interest in comprehensive research to establish the extent and patterns of exposure. The Tasmanian wedge‐tailed eagle (*Aquila audax fleayi*) is a subspecies endemic to the island of Tasmania. The population is listed as endangered at both state and federal levels (State Government of Tasmania 1995; Commonwealth of Australia 1999), with conservation concern based on a series of known threats, including nest failures caused by anthropogenic disturbance, low rates of breeding success, habitat loss, collisions with anthropogenic structures, and illegal persecution (Mooney and Holdsworth 1991; Mooney 1997; Bell and Mooney 1998; Department of Primary Industries, Parks, Water and Environment 2006). However, despite its behavioral susceptibility and the potentially large amount of ingestible lead present in Tasmania, there has been no formal research into the degree to which these birds are lead exposed. To investigate the extent and routes of lead exposure in Tasmanian wedge‐tailed eagles, we 1) assessed the concentrations of lead in different tissues; 2) tested for age‐specific differences in lead concentrations; 3) tested for seasonal changes in lead exposure; and 4) assessed patterns of lead isotope ratios in tissues and determined the similarity of these ratios to those of lead‐based bullets.

## MATERIALS AND METHODS

### Study location

**Figure 1:**
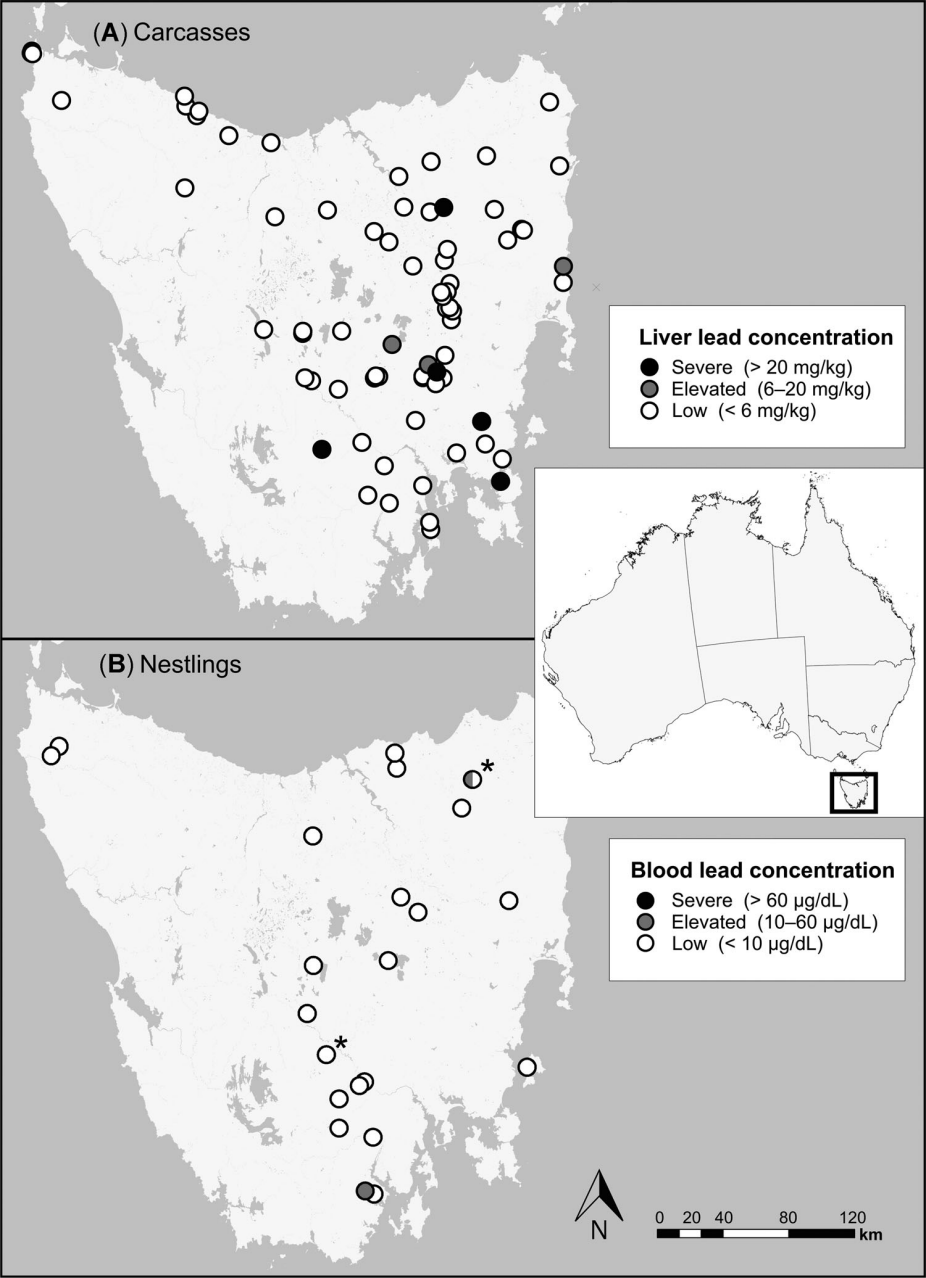
Location and diagnostic categorization of (**A**) liver lead concentrations from 109 carcasses of free‐flying Tasmanian wedge‐tailed eagles that died between 1996 and 2018, and (**B**) blood lead concentrations of 24 nestlings of the same species that were sampled live during 2017 and 2018. Location was recorded for 72 of the carcasses and all nestlings. Nestlings were sampled from the same nest in consecutive seasons at 2 nest sites (indicated by asterisks).

Tasmania is an island state of Australia located 240 km south of the Australian mainland, covering a land area of 68 150 km². The separation of Tasmania from mainland Australia for approximately 10 000 yr has resulted in an array of endemic flora and fauna (Stattersfield et al. 1998; De Salas and Baker 2014; Department of Primary Industries, Parks, Water and Environment 2014). Eagles from much of Tasmania were sampled, enabling an assessment of lead contamination across the population (Figure 1).

### Sample collection and preparation

Our samples were from 2 sources. First we collected liver and bone from eagle carcasses (broadly characterized as adults and pre‐adults based on plumage; Supplemental Data, S1). Second, we drew blood from live‐caught nestlings. The present study was conducted in accordance with the University of Tasmania Animal Ethics Committee Permit A0015930 and Department of Primary Industries, Parks, Water and Environment scientific permits TFA17127 and TFA17328.

Carcasses had been collected opportunistically by government departments, industry representatives, and volunteers, between 1996 and 2018, and stored frozen by the Department of Primary Industries, Parks, Water and the Environment (Threatened Species Section, Hobart, TAS, Australia) and the Tasmanian Museum and Art Gallery (Collection and Research Facility, Rosny, TAS, Australia). Between May 2017 and March 2018, we defrosted carcasses and harvested tissue. At necropsy, we collected a liver lobe, sectioned a femur diaphysis (≈3 cm length from the middle of the femur), and estimated the bird's age at death.

Nestlings were sampled prior to fledging (estimated age 65–75 d) during 2017 and 2018. We selected target nests identified during fixed‐wing aerial nest surveys (Forest Practices Authority 2014). The age of nestlings, assessed on plumage development, was recorded during the surveys to guide timing of our visit to the nest sites. There was never more than one nestling present in a nest in any year; at 2 nests we sampled a single bird in each of 2 consecutive years (Figure 1B). From each nestling we took approximately 2 mL of whole blood from the brachial vein using a 26‐gauge needle. We stored blood in low‐lead Vacutainers (Beckton Dickinson), which were refrigerated for 1 to 4 d until they could be transported to a freezer.

Blood samples and necropsied tissues were stored at −20 °C until sample preparation. We thawed all samples to room temperature for preparation. We processed each liver and femur sample with new gloves and scalpel blades, and we washed forceps beforehand. We removed all adherent nontarget tissues (e.g., muscle, connective tissue, marrow) from each sample using a stainless‐steel scalpel blade. We then sectioned target tissue from the middle of each liver lobe (≈1.3 g) and femur diaphysis (≈460 mg). We dehydrated femur and liver samples at 60 °C until a constant weight was reached. Dried samples were weighed on a digital balance (precision ± 0.0001 g; Mettler Toledo). A 1‐mL volume of each blood sample was also dehydrated and weighed using the same method. We stored samples in metal‐free plastic containers until digestion.

### Lead concentration and isotope analysis

Samples were analyzed for lead concentration and isotope ratios at Edith Cowan University Analytical Facility (Joondalup, WA, Australia). Liver and bone samples were homogenized, and 0.4 g was aliquoted into inductively coupled plasma (ICP)‐grade Teflon vessels containing 5 mL of trace analysis–grade nitric acid 68% (Primer Plus™; Fisher Scientific), 0.5 mL of trace metal–grade hydrochloric acid 34 to 37% (Fisher Scientific), and 3 mL of hydrogen peroxide 30% (Emsure ISO®; Merck). Samples were digested for 15 min in a Multiwave GO microwave digestion system (Anton Paar) set to 150 °C. After digestion, samples were diluted to 50 mL with Milli‐Q® reverse osmosis deionized water and transferred to polypropylene tubes for analysis. Blood samples were prepared by a similar method using digestion in 2 mL of nitric acid, 0.2 mL of hydrochloric acid, and 1 mL of hydrogen peroxide. Blood sample solutions were then sonicated at 60 °C for 1 h and diluted to 10 mL using Milli‐Q® reverse osmosis deionized water.

Lead concentrations were determined via ICP–mass spectrometry (MS) using an iCAP Q ICP‐MS (Thermo‐Fisher Scientific) coupled to an ASX‐520 AutoSampler (Agilent Technologies). Data acquisition, element quantitation, and isotope percentage analyses were carried out using Qtegra (Thermo‐Fisher Scientific). The instrument was calibrated using concentration ranges of iCAPQ element standards (Thermo‐Fisher Scientific) and ICP–MS‐68A solutions (High Purity Standards) to provide standard curves before analysis. Certified reference materials (CRMs) were used as positive controls for each tissue type. These were Bone Ash Standard Reference Material 1400 (National Institute of Standards and Technology) and Bovine Liver Certified Reference Material BCR–185R (Institute for Reference Materials and Measurements). Two digestions were carried out on each CRM with 2 ICP–MS readings of each digestion. Accuracy of CRM ICP–MS readings averaged 96.7% for femur and 104.1% for liver. Every 10th sample was re‐analyzed for a duplicate read (average relative standard deviation [RSD] 1.8%), and duplicate blind sample digestions were carried out for 20 randomly selected samples (average RSD 5.5%). Lead concentrations were measured as mg/kg dry weight. Limits of quantification and limits of detection (LOD) for the analysis were 0.005 and 0.0015 mg/kg, respectively.

Lead isotopes were determined as counts per second (cps) measured at m/z 204, 206, 207, and 208. Isotope readings were adjusted according to readings from a calibration lead solution at m/z 204 (1.40%), m/z 206 (24.10%), m/z 207 (22.10%), and m/z 208 (52.40%). Analyses of isotope data focused on the lead^207/206^ ratio. This ratio is used most commonly in research investigating sources of lead exposure in birds (Finkelstein et al. 2012; Behmke et al. 2015; Katzner et al. 2017).

### Interpretation of lead concentrations

To interpret liver lead concentrations in terms of their potential physiological effects, we used previously identified thresholds of <6 mg/kg dry weight as evidence of low levels of exposure with limited health implications (Pain et al. 1995; Franson 1996), 6 to 20 mg/kg dry weight as elevated with some health implications, and >20 mg/kg dry weight indicative of severe exposure, representing a likely lethal dose (Pain et al. 1995).

We used an exposure threshold of femur lead concentrations <10 mg/kg dry weight as indicative of low exposure (reviewed in Franson and Pain 2011), 10 to 20 mg/kg dry weight as elevated, and concentrations >20 mg/kg dry weight as severe (bone lead concentrations >20 mg/kg dry wt have been observed after lethal poisonings in raptors; Rodriguez‐Ramos Fernandez et al. 2011; Jenni et al. 2015). However, the long‐term accumulation of lead in bone, the potential for recirculation of lead from bone to blood, and the difficulty of sampling bone from living birds complicate the inference of physiological responses to these concentration thresholds.

To compare our blood lead concentrations with those in other studies, we converted dry blood results (provided in mg/kg dry wt) to wet weight (μg/dL) by multiplying the dry weight concentrations by the dry to wet weight ratios of each sample. We used an exposure threshold of blood lead <10 μg/dL as indicative of low exposure (Finkelstein et al. 2012), although we recognize that both physiological and behavioral effects have been reported at levels well below this (e.g., Ecke et al. 2017). Blood lead concentrations of 10 to 60 μg/dL were considered elevated, and concentrations >60 μg/dL were considered severe (Fallon et al. 2017).

### Data analysis

We calculated standard summary statistics (arithmetic mean, arithmetic standard deviation [SD], geometric mean [GM], geometric SD, and median) for liver and femur lead concentrations of all samples and also for each age class. In the case of the blood lead concentrations, for which some samples had values below the LOD of the ICP–MS (i.e., nondetects), we used a Kaplan–Meier cumulative probability distribution (cenfit; R package NADA; Lee, 2017) to calculate summary statistics.

We used separate statistical analyses to address each of our research questions. We did this to maximize the data available to address each question. Despite the utility of mixed‐effects models, they are not well suited to datasets with large numbers of missing values (Nakagawa and Freckleton 2011). This was particularly relevant to our data because most of the eagle carcasses we considered lacked certain metadata (e.g., they were missing date and/or location of carcass recovery). That said, to address potential concerns about spurious results from running many tests, we compared our results with multiple linear regression models when possible, and we report the analyses (question‐specific or mixed‐model) that were most informative. When tissue lead data did not meet assumptions for parametric univariate analyses, we used nonparametric equivalents. We used a significance level of α = 0.05 in all analyses.

Eagle carcasses were not collected, nor were nestlings identified and sampled, in a spatially random manner. Thus we used Mantel tests (mantel; R package ecodist; Goslee and Urban 2020) and multiple regressions on distance matrices (MRM; R package ecodist) with ranked correlation distances (Spearman) to check for evidence of spatial clustering of samples in relation to lead tissue levels. Significance of MRM coefficients and *R* ^2^ values were calculated using 1000 permutations. Significance of the Mantel tests was assessed using the H_0_ of no spatial correlation (negative or positive) between tissue lead levels and the distance between samples.

To investigate differences between femur and liver lead concentrations, we used a Wilcoxon signed rank test (wilcox.test; R Development Core Team 2016) and the Kendall's tau statistic to measure the correlation between liver and femur concentrations (cor test; R Development Core Team 2016). To understand age‐specific differences in tissue lead concentrations (our second research objective), we used Wilcoxon rank sum tests to compare lead concentrations in liver and femur between the 2 different age groups. To test for seasonal changes in lead exposure (our third research objective), we used a Kruskal–Wallis rank sum test (kruskal test; R Development Core Team 2016) to compare lead concentrations in liver among seasons. We used a multiple linear regression to jointly assess the effect of both age and season on liver lead concentrations using a reduced dataset of those birds for which we had age and season data. For this model, liver lead concentrations were log10‐transformed to meet distributional assumptions of the statistical test more closely.

Finally, we investigated isotopic patterns of lead exposure (our fourth research objective) by assessing differences in lead^207/206^ ratios. We first assessed variation in lead^207/206^ ratios between liver samples with elevated lead concentrations (>6 mg/kg) and those with low concentrations (<6 mg/kg) using a modified signed‐likelihood ratio test (mslr_test; R package cvequality; Marwick 2019). After concluding equal variance, we used a Wilcoxon rank sum test to compare isotope ratios between these 2 groups. We used the Kendall's tau statistic to measure, separately for liver and for blood, the correlation between lead isotope ratios and lead concentrations. We plotted data with the Theil–Sen estimator line to visualize correlations. To establish whether ammunition was a likely source of exposure, we compared the overlap in tissue lead^207/206^ isotope ratios with those for lead‐based ammunitions (following methods in Behmke et al. 2015 and Finkelstein et al. 2012). Sampling local ammunition was not feasible during the present study, and so for comparison we used published lead^207/206^ isotope ratios (Sjåstad et al. 2014) for 10 brands of ammunition available in Tasmania (Supplemental Data, S2). We did not assess patterns in lead^207/206^ ratios in bone samples, because the long‐term accumulation of lead in bone (Fisher et al. 2006) means that its isotope ratios likely reflect lead from multiple sources and exposure events.

## RESULTS

We collected tissue from 109 eagle carcasses, of which 27 were adults and 82 pre‐adults. We analyzed tissue lead concentrations in 108 femur samples (26 adults and 82 pre‐adults) and 106 liver samples (26 adults and 80 pre‐adults). The location of carcass recovery was recorded for 72 eagles, and the date of recovery was recorded for 61. We collected blood samples from 24 eagle nestlings. There was no evidence of spatial organization in lead concentrations of liver (*n* = 71; MRM: *R* ^2^ = 0.003, *F* = 8.382, *p* = 0.393; Mantel: *p* = 0.721, *r* = –0.029), femur (*n* = 72; MRM: *R* ^2^ = 0.001, *F* = 2.575, *p* = 0.574; Mantel: *p* = 0.473, *r* = –0.053), or nestling blood samples (*n* = 24; MRM: *R* ^2^ < 0.001, *F* = 0.097, *p* = 0.898; Mantel: *p* = 0.68, *r* = –0.054).

### Extent of lead exposure

**Figure 2:**
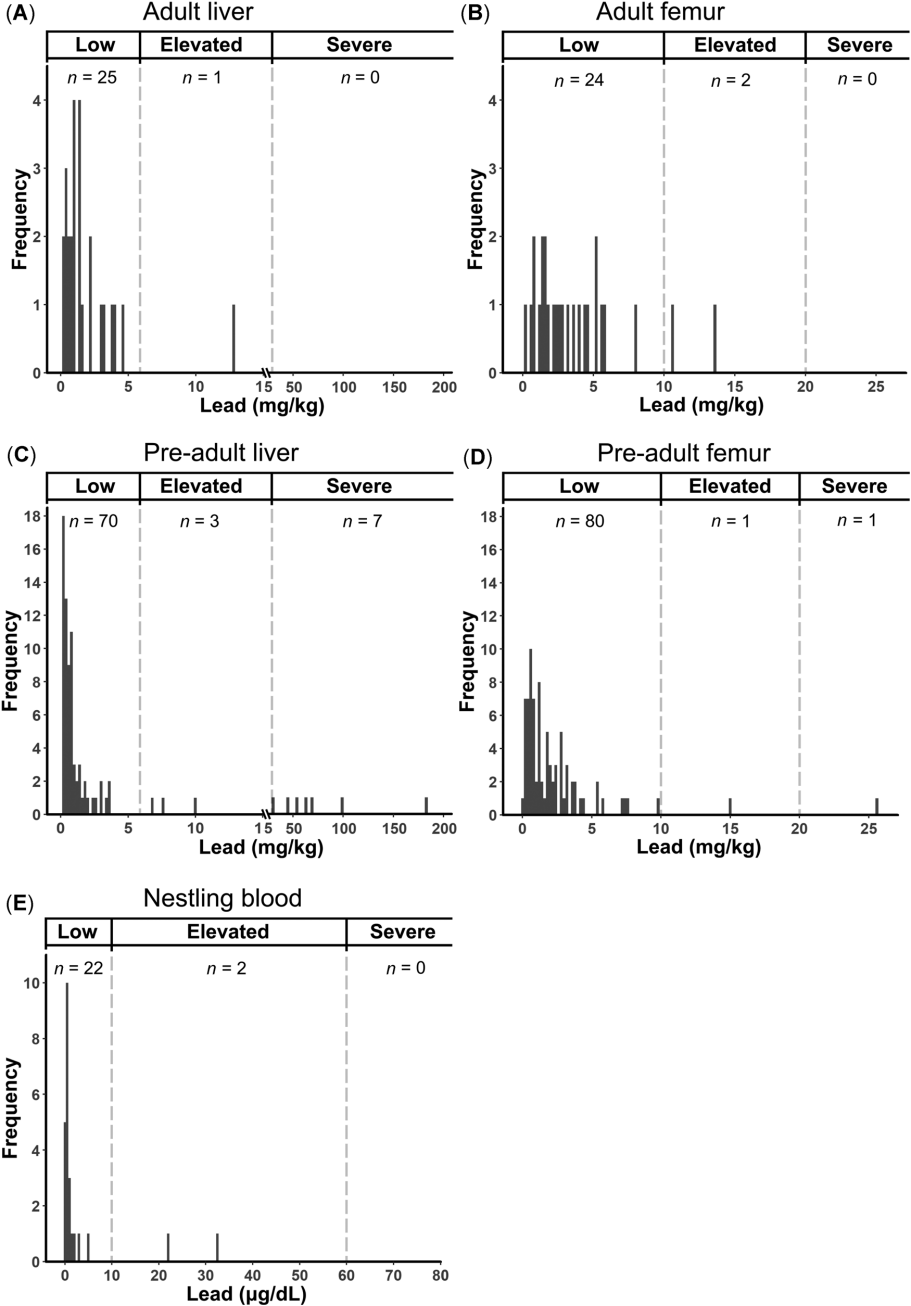
Tissue lead concentrations for Tasmanian wedge‐tailed eagles of different age classes that died between 1996 and 2018 (free‐flying adults and pre‐adults) or were sampled live during 2017 and 2018 (nestlings). Each frequency histogram shows the number of adult (**A**) liver and (**B**) femur, pre‐adult (**C**) liver and (**D**) femur, and (**E**) nestling blood samples within 0.2 mg/kg dry weight (liver or femur) or 0.04 μg/dL wet weight (blood) bins. The threshold values indicative of low, elevated, and severe lead poisoning are shown by the dashed lines. The number of individuals falling within each threshold is noted. Liver lead plots (**A**) and (**C**) are presented with a break in the *x* axis for graphical representation of the data.

Lead concentrations were above the LOD of the ICP–MS in all femur and liver samples analyzed (femur: 1.514 ± 3.106 mg/kg dry wt [GM ± SD] and 2.708 ± 3.484 [$\bar{x}$ ± SD]; liver: 1.032 ± 4.492 and 6.393 ± 22.611; Table 1). Liver lead concentrations were elevated (6–20 mg/kg) in 3.8% of the samples (one adult and 3 pre‐adults) and severe (>20 mg/kg) in 6.6% (7 pre‐adults; Figure 2A and C). Femur lead concentrations were elevated (10–20 mg/kg) in 2.8% of the samples (2 adults and one pre‐adult) and indicative of severe poisoning (>20 mg/kg) in 0.9% (one pre‐adult; Figure 2B and D).

**Table 1:** Age‐specific summary statistics describing tissue lead concentrations for wedge‐tailed eagles in Tasmania^a^

Tissue	Age	No.	<LOD	Median	GM	SD	Range
Femur	Adults	26	0	2.69	2.51	2.60	0.20–13.69
(mg/kg; dry wt)	Pre‐adults	82	0	1.22	1.29	3.14	0.06–25.57
	Total	108	0	1.73	1.51	3.11	0.06–25.57
Liver	Adults	26	0	1.21	1.23	2.61	0.23–12.72
(mg/kg; dry wt)	Pre‐adults	80	0	0.68	0.97	5.16	0.10–181.60
	Total	106	0	0.75	1.03	4.49	0.10–181.60
Blood (μg/dL)	Nestlings	24	1	0.51	3.08	7.73	<LOD–32.74

^a^ Femur and liver samples were taken from eagle carcasses collected between 1996 and 2018. Blood samples were collected from live nestlings during 2017 and 2018. Geometric means (GM) and standard deviations (SD) are presented for femur and liver samples. Summary statistics for nestling blood were calculated using a Kaplan–Meier cumulative probability distribution to account for lead concentrations below the limit of detection (LOD). Femur and liver lead concentrations are reported on a dry weight basis. Blood concentrations are reported on a wet weight basis.

Lead concentrations in femur samples were significantly higher than in liver samples (*Z* = 1564, *p* < 0.001, *n* = 105). However, the 6 highest liver lead concentrations (range: 43.9–181.6 mg/kg) were markedly higher than the highest femur lead concentration (25.6 mg/kg). Lead concentrations in femur and liver samples from the same individual were weakly correlated with each other (tau = 0.438, *p* < 0.01, *z* = 6.623; Supplemental Data, Figure S3).

We detected lead concentrations above the LOD (0.15 μg/dL) in 23 of 24 nestling blood samples (3.083 ± 7.731 μg/dL [$\bar{x}$ ± SD]; range: <LOD–32.74 μg/dL; Table 1). Lead concentrations of 2 nestlings (8.3%) were elevated (10–60 μg/dL; Figure 2E), and none showed evidence of severe exposure (>60 μg/dL). One of the 2 nestlings with an elevated blood lead concentration was from a nest sampled in 2 yr; however, the nestling sampled in the other year from that nest did not have an elevated concentration.

### Differences between age groups & seasons

**Figure 3:**
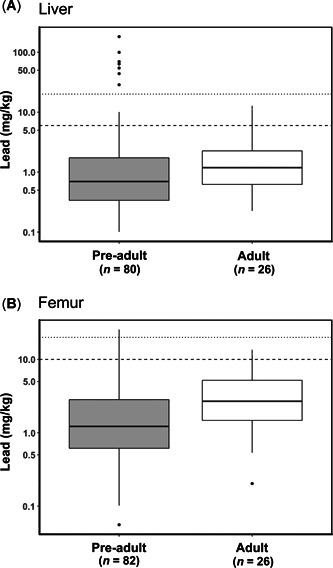
Box plots of (**A**) liver lead concentrations and (**B**) femur lead concentrations for adult and pre‐adult Tasmanian wedge‐tailed eagles that died between 1996 and 2018. Plots are presented on a log scale for graphical representation of the data. The horizontal lines represent lead exposure thresholds (values above the dashed lines indicate elevated tissue lead concentrations, and values above the dotted lines indicate severe tissue lead concentrations). Box plot whiskers are extended to maximum values within 150% of the interquartile range; values beyond this are plotted individually as outliers. Tissue lead concentrations are on a dry weight basis.

Lead concentrations in livers were similar in both age groups (*W* = 1307, *p* = 0.05; Figure 3A). However, the proportion of birds with elevated or severe liver lead levels (>6 mg/kg) was higher for pre‐adults (12.5%, *n* = 10) than adults (3.9%, *n* = 1). Lead concentrations of femurs from adult birds were significantly higher than those from pre‐adult birds (*W* = 1454, *p* = 0.005; Figure 3B). The proportion of birds with elevated or severe femur lead levels (>10 mg/kg) was also higher for adults (7.7%, *n* = 2) than for pre‐adults (2.4%, *n* = 2). However, the only bird with femur lead concentrations indicative of severe exposure (>20 mg/kg) was a pre‐adult.

We detected no interseasonal difference in liver lead concentrations (spring: 2.264 ± 5.636 mg/kg dry wt [GM ± SD], *n* = 11; summer: 1.772 ± 7.04, *n* = 14; autumn: 0.763 ± 1.935, *n* = 10; winter: 1.149 ± 5.728, *n* = 25; *χ* ^*2*^ = 3.55, *df* = 3, *n* = 60, *p* = 0.314; Supplemental Data, Figures S4 and S5). That said, elevated and severe liver lead concentrations were only recorded in winter, spring, and summer, and never in autumn.

We ran a linear model for the 60 samples for which we had data on both season of death and age at death. Results of this analysis were comparable to those of the 2 Wilcoxon tests (Supplemental Data, Table S2). Because the sample size was smaller in this test and because residuals indicated poor matching to distributional assumptions, we interpreted only the Wilcoxon tests.

### Isotopic patterns in exposure

**Figure 4:**
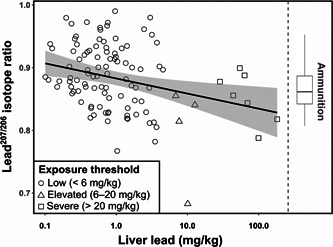
Relationship between the lead^207/206^ isotope ratio and the concentration of lead in liver tissue. The *x* axis is presented on the log scale. The Theil‐Sen estimator line is presented with the 95% confidence interval indicated by the shaded area. The range in published isotope ratios (Sjåstad et al. 2014) of lead‐based ammunition brands available in Tasmania is shown on the right.

Mean lead^207/206^ isotope ratios within liver samples were 0.8835 ± 0.0539 (±SD; range: 0.6829–0.9901; *n* = 106). The lead^207/206^ ratios of eagles with elevated hepatic lead concentrations (>6 mg/kg, *n* = 11) were significantly different from those with low lead concentrations (<6 mg/kg, *n* = 95; *W* = 802, *p* = 0.004; Supplemental Data, Figure S6), despite the disparity in sample size, which reduces power to detect a difference. The level of variation in lead^207/206^ isotope ratios was similar between these 2 groups (modified signed likelihood ratio test = 0.726, *p* = 0.394). Lead^207/206^ isotope ratio was negatively correlated with liver lead concentration (tau = –0.212, *p* < 0.002, *z* = –3.216; Figure 4). One liver sample with elevated lead levels (6–20 mg/kg) had a very low lead^207/206^ ratio. To assess the influence of this sample, we repeated analyses with this sample removed, which resulted in similar comparative (*W* = 707, *p* = 0.012) and correlative conclusions (tau = –0.199, *p* < 0.003, *z* = –3.015). For nestling blood samples with lead (*n* = 23), lead^207/206^ ratios were not correlated with blood lead levels (tau = –0.257, *p* = 0.09, *T* = 94; Supplemental Data, Figure S7). The low number of nestling blood samples with elevated lead levels (*n* = 2) precluded statistical comparisons of lead^207/206^ ratios between different blood lead thresholds.

Liver lead^207/206^ isotope ratios overlapped the published range of lead^207/206^ ratios for a subset of brands of lead‐based bullets available in Tasmania (0.8070–0.9561). The majority (72.7%) of liver samples with elevated lead concentrations (>6 mg/kg; *n* = 11) had lead^207/206^ ratios that were within this reported isotopic range of ammunition. The majority (91.3%) of nestling blood samples with detected lead (*n* = 23) also had lead^207/206^ ratios that were within the published isotopic range of ammunition brands available in Tasmania.

## DISCUSSION

Our study provides the first comprehensive data on lead exposure of an Australian raptor species and highlights the value of analyzing different tissues when assessing the extent of lead contamination. Whereas a low proportion of femur samples had elevated lead concentrations, liver concentrations showed more substantial evidence of lead exposure. The higher lead concentrations detected in femurs of older birds compared with femurs from pre‐adults suggests that birds are chronically exposed. The absence of seasonal trends in hepatic lead suggests that risk of exposure may not change throughout the year. Lastly, the isotope ratios are consistent with an ammunition source of contamination, but additional information on the isotope ratios of other lead sources would be useful to clarify routes of exposure in Tasmania.

### Extent and demographic patterns of lead exposure

The femur lead concentrations we detected in the Tasmanian wedge‐tailed eagle spanned a narrower and lower range than those of other raptors on other continents (e.g., European golden eagles [*Aquila chrysaetos*; 0.4–54.21 mg/kg, *n* = 46; Ganz et al. 2018], black vultures [*Coragyps atratus*; 4.5–540 mg/kg, *n* = 98; Behmke et al. 2015], and Spanish imperial eagles [*Aquila adalberti*; <LOD–41.68 mg/kg, *n* = 84; Rodriguez‐Ramos Fernandez et al. 2011]). The mean femur lead concentrations we recorded were similar to those found in preliminary work on wedge‐tailed eagles in Western Australia (3.14 mg/kg ± 3.9; Lohr et al. 2020), although the maximum concentration we report is dramatically higher than that in the small sample they considered (10.2 mg/kg, *n* = 11). The maximum bone lead concentrations we detected are similar to concentrations observed in studies of golden and bald eagles (*Haliaeetus leucocephalus*; 18 mg/kg, *n* = 49; Wayland et al. 1999) and Egyptian vultures (*Neophron percnopterus*; 30 mg/kg, *n* = 39; Gangoso et al. 2008). The proportion of birds with severe bone lead concentrations (>20 mg/kg) was lower in the Tasmanian wedge‐tailed eagle (2%) than that reported for European golden eagles (30%, *n* = 46; Ganz et al. 2018), American golden eagles (29%, *n* = 17; Jenni et al. 2015), white backed vultures (*Gyps africanus*; 6%, *n* = 18; van den Heever et al. 2019), and Egyptian vultures (4%, *n* = 39; Gangoso et al. 2008).

Although these patterns could be interpreted to suggest limited chronic lead exposure of Tasmanian wedge‐tailed eagles, such a conclusion is likely premature. Instead, because pre‐adult birds have not lived long enough to exhibit signs of chronic exposure, it may be that the patterns in our data are driven by the predominance of pre‐adult birds in our study. Pre‐adults represented 76% of our sample, a notably higher proportion than in other studies assessing bone lead concentrations of raptors (e.g., 40% [Wayland et al. 1999], 44% [Madry et al. 2015] 44% [Ganz et al. 2018], and 49% [Gangoso et al. 2008]) or than would be expected from random sampling of a population of long‐lived eagles (Katzner et al. 2006). Bone lead concentrations reflect lifetime exposure due to the accumulation over time of lead in bone tissue (Fisher et al. 2006). Older birds of many species therefore display higher bone lead concentrations, an indicator of repeated exposure to sublethal levels of lead (Gangoso et al. 2008; Rodriguez‐Ramos Fernandez et al. 2011; Ganz et al. 2018). Our detection of an age‐related increase in femur lead burdens in the population of Tasmanian wedge‐tailed eagles suggests that these birds also are repeatedly exposed to lead. The high proportion of pre‐adult birds included in our study sample therefore impedes both comparisons with femur concentrations in other raptor studies and our ability to draw inferences in terms of population‐wide femur lead levels.

The concentrations of lead that we detected in the liver samples are suggestive of frequent, widespread lead exposure of Tasmanian wedge‐tailed eagles. This is consistent with the detection of an age‐related increase in femur lead concentrations. The median liver lead concentration we measured (0.735 mg/kg) lies within the range of estimates for other avian scavengers threatened by lead poisoning (0.23–1.38 mg/kg; Rodriguez‐Ramos Fernandez et al. 2011; Berny et al. 2015). Furthermore, recent studies using opportunistic sampling of dead and moribund avian scavengers generally include a proportion of birds with no detectable liver lead (Carneiro et al. 2014; Warner et al. 2014; Jenni et al. 2015; Ganz et al. 2018). Although the differing LODs complicate interstudy comparisons, the presence of lead in every single liver sample suggests that lead exposure is widespread in the Tasmanian wedge‐tailed eagle population.

The proportion of birds we detected with severe hepatic lead concentrations (6.6% were >20 mg/kg) is higher than those found in congeners (0–5.5%; Rodriguez‐Ramos Fernandez et al., 2011; Jenni et al. 2015; Madry et al. 2015; Ganz et al. 2018). In fact, we could find only one report of an individual liver lead concentration higher than the 181.6 mg/kg maximum detected in our study (243 mg/kg; Wayland et al. 1999). Such high levels of lead exposure may reflect both 1) the large amount of anthropogenic lead in the Tasmanian landscape, and 2) that the unique life history characteristics of wedge‐tailed eagles increases their risk of exposure, in that they appear to scavenge more than congeneric species.

The high proportion of birds with severe hepatic lead concentrations may also explain the lower lead concentrations we detected in the femur samples. The threshold we used to indicate severe exposure (>20 mg/kg dry wt) represents a likely lethal dose (Pain et al. 1995). An alternative explanation for the pattern we detected is that a high proportion of birds is being acutely exposed and dying before bone lead levels have time to reach high levels.

A very high proportion (95%) of nestlings we tested had been exposed to lead. Although this rate is higher than that reported for other raptors (Harmata 2011; Katzner et al. 2017; Bruggeman et al. 2018; Herring et al. 2020), that most exposure was at a low level is consistent with findings for other raptors (Carlson et al. 2012). Nestling raptors can be exposed to lead from inhalation, ingestion, or maternal transfer (Pattee 1984; Katzner et al. 2017; Bruggeman et al. 2018). Wedge‐tailed eagles, like other raptors, are thought to mainly provision their nestlings with live‐caught prey (Olsen 2005); however, exposure through consumption is still possible due to prey species surviving being shot and containing lead ammunition (Pain et al. 2015). Similarly, the high blood lead concentrations found in 2 nestlings are indicative of ingestion, rather than inhalation or maternal transfer (Katzner et al. 2017), and therefore suggest that adult Tasmanian wedge‐tailed eagles do bring lead‐contaminated food to their young (be it scavenged or live caught).

### Seasonal patterns in lead exposure

Although our carcass sampling was opportunistic, the absence of seasonal differences in lead exposure of Tasmanian wedge‐tailed eagles likely reflects the near absence of seasonality in local ammunition use. An increase in lead exposure during designated hunting seasons has been reported in scavenging bird populations in Europe (Ecke et al. 2017), Africa (Garbett et al. 2018), Canada (Legagneux et al. 2014), and the United States (Bedrosian et al. 2012; Lindblom et al. 2017). In the regions where these studies were conducted, there are legally defined hunting seasons that result in substantial temporal trends in the numbers of animals that are shot and thus in lead availability in the landscape. In contrast, Tasmanian legislation allows shooting throughout the year (Tasmanian Government 2002), thereby minimizing seasonal fluctuations in availability of carcasses and lead.

### Ammunition as a potential source of lead exposure

The range in isotope ratios that we detected was much greater than those documented for other avian scavengers (Finkelstein et al. 2012; Behmke et al. 2015; Mateo‐Tomás et al. 2016). This increased range could be due to the wider variety of ammunition brands available in Australia, and to sources of lead contamination other than ammunition. Ammunition in Tasmania comes from at least 3 different continents (Supplemental Data, S2), meaning that lead isotope ratios there are likely broader than would be the case for continents where ammunition is locally made. Moreover, in Tasmania there are potential sources of anthropogenic lead for which we do not have isotopic information (e.g., mining, paint, coal emissions, fuels). There can also be overlap in the isotopic ratios of different sources of anthropogenic lead found in a region, making inferences on sources of exposure difficult without local isotopic information (Finkelstein et al. 2012; Behmke et al. 2015; Berny et al. 2015). Expanded isotopic analyses of environmental and anthropogenic lead sources in Tasmania would help to clarify exposure pathways.

Our ability to link the lead exposure detected in the Tasmanian wedge‐tailed eagle to lead‐based ammunition was limited in 3 ways. First, although recent work has frequently used Q ICP–MS to measure lead^207/206^, there are concerns over the precision of lead^207/206^ estimates obtained by this method (Gulson et al. 2018). Second, we were unable to directly measure lead isotopes in ammunition available in Tasmania, instead relying on published isotope ratios for ammunition brands currently available in the region. The duration over which the eagle carcasses in our study were collected (22 yr) also complicates this issue because lead isotope ratios for different brands of ammunition may change over time (particularly with contemporary ammunition often manufactured using recycled lead; Koons and Grant, 2002). Third, we were only able to make isotopic comparisons with lead‐based bullets. Lead shotgun shot can also be a source of contamination for raptors (see Donázar et al. 2002; Helander et al. 2009), and is therefore a future research need for the wedge‐tailed eagle.

Despite the ambiguity of our isotopic data, they do provide some evidence that at least some of the lead exposure we recorded arose from consumption of ammunition. This is because, first, the proportion of individuals with lead^207/206^ isotope ratios within the range of ammunition was comparable to other studies implicating ammunition as a source of lead poisoning (e.g., 79%; Finkelstein et al. 2012). Second, we detected lower isotope ratios in more highly exposed birds, patterns similar to those found for other avian scavengers (Church et al. 2006; Finkelstein et al. 2012; Legagneux et al. 2014). These lead isotope shifts in acutely exposed birds can be due to the lower lead^207/206^ ratios associated with ammunition (Church et al. 2006; Finkelstein et al. 2012; Legagneux et al. 2014).

### Conservation implications

The physiological effects of different levels of lead exposure can be difficult to interpret because of the paucity of experimental evidence quantifying the effects of tissue lead concentrations. Lead concentrations we have categorized as “low” do not equate to no physiological effect (Ganz et al. 2018). Instead, the thresholds used for low exposure are mostly based on a lack of apparent, usually visible, symptoms. However, the strong selection pressure in wild animals to hide signs of illness risks underestimating the effects. Furthermore, lead has been shown to have physiological effects even at low concentrations (Lanphear et al. 2005; Espín et al. 2015; Herring et al. 2020), suggesting that even low exposure may be of concern.

Our results indicate that lead exposure is likely a significant factor to consider in conservation management for the Tasmanian wedge‐tailed eagle. Although some Australian eagles are known to die directly from lead poisoning (Mooney 1986), lead may negatively affect the population in other ways. For example, lead at low concentrations may increase the susceptibility of individuals to other causes of mortality, such as collisions with anthropogenic structures and vehicles (Kelly and Kelly 2005; Golden et al. 2016; Ecke et al. 2017). Similarly, lead poisoning of nestlings may lead to reproductive failure, and thus contribute to the low fledging success rate that is reported for the Tasmanian wedge‐tailed eagle population (Department of Primary Industries, Parks, Water and Environment 2006; Forest Practices Authority 2017). Additional work focused on nestlings that die before fledging may provide insights into this concern.

Our study highlights the importance of further research and efforts to address lead contamination throughout the ecosystem and in other Australian regions. In Tasmania, high levels of lead exposure are unlikely to be confined to wedge‐tailed eagles. In fact, numerous species are known to scavenge shot carcasses, including the endangered Tasmanian devil (*Sarcophilus harrisii*; Hivert et al., 2018; International Union for Conservation of Nature 2020). However, there is a paucity of research on the ecological effects of lead‐based ammunition in Australia (Hampton et al. 2017). In the United States and Europe, the environmental risks of lead‐based ammunition have been recognized, and lead‐based ammunition is being increasingly restricted (Golden et al. 2016; Ganz et al. 2018; European Chemicals Agency 2019). Reductions in the use of lead‐based ammunition can be effective in reducing exposure rates, as shown by the fact that blood lead concentrations in scavenging bird species in California significantly declined after localized bans on lead‐based ammunition were implemented in 2008 (Kelly et al. 2011). If lead contamination from spent ammunition is pervasive in Australia, then equivalent mitigative efforts are likely to be effective in reducing the exposure levels of at‐risk species throughout the country.

## Supplemental Data

The Supplemental Data are available on the Wiley Online Library at https://doi.org/10.1002/etc.4914.

## Disclaimer

Any use of trade, product, or firm names is for descriptive purposes only and does not imply endorsement by the US Government.

## Author Contributions Statement

J. Pay, T. Katzner, J. Wiersma, C. Hawkins, A. Koch, and E. Cameron conceived the study. W. Brown and N. Mooney collected carcasses. J. Pay collected tissue samples from carcasses. J. Wiersma collected blood samples. J. Pay prepared all samples for laboratory analysis. J. Pay analyzed the data with assistance from T. Katzner. J. Pay led writing of the manuscript, and all co‐authors contributed to revisions of the manuscript.

## Supporting information

This article includes online‐only Supplemental Data.

Supporting information.

## Data Availability

Data, associated metadata, and calculation tools are available from the corresponding author (James.Pay@utas.edu.au).
