# Supplementary material for: High Frequency of Lead Exposure in the Population of an Endangered Australian Top Predator, the Tasmanian Wedge‐Tailed Eagle (Aquila audax fleayi)
Source: Environ Toxicol Chem. 2020 Dec 3;40(1):219–30. doi: 10.1002/etc.4914 (PMC7839751; doi:10.1002/etc.4914)
Supplement: Supplementary file 1 — Supporting information. [file ETC-40-219-s001.docx]

Supplementary material: **High frequency of lead exposure in the population of an endangered Australian top predator, the Tasmanian wedge-tailed eagle (*Aquila audax fleayi*)**

Appendix A

*Age categories*

Tasmanian wedge-tailed eagle carcasses were categorized into two age classes (pre-adult and adult) based on plumage differences. Pre-adults are estimated to include all birds younger than their 5^th^ year (Ridpath and Brooker, 1986). Plumage characteristics used to identify pre-adult eagles were a pale nape, broad dorsal wing bars (more than quarter of wing width; Debus, 2012), barring apparent on remiges and rectrices, pale tail coverts, and light brown elements to back feathers (Debus, 2012; Olsen, 2005; Ridpath and Brooker, 1986 see Figure A.1). Birds categorized as adults had darker plumages (particularly on the back, chest, nape, and neck), narrower dorsal wing bars (less than quarter of wing width; Debus, 2012), brown tail coverts, and a pale base to flight feathers (Debus, 2012; Ridpath and Brooker, 1986; see Figure A.2).

A more accurate method to determine age using a molt chart (e.g. Bloom & Clark, 2001) has not been developed for the species. In lieu of a molt chart not being available we used the plumage characteristics described above. However, there are some important considerations with this aging method. First, pre-adult plumage can remain into the year that eagles become mature. Likewise, population stability (i.e. levels of unnatural mortality and the number of non-territorial, non-breeding adults) can influence the age at which birds breed (Bell and Mooney, 1998; J. Wiersma pers. comm). Second, without a molt chart, separating pre-dispersal juveniles from older pre-adults is subjective. Therefore, we used the two age classes described, representing birds definitely of breeding age (adults) and all younger birds (pre-adults).

**References**

Bell, P., & Mooney, N., (1998). *Wedge-tailed Eagle Recovery Plan 1998-2003*. Department of Primary Industries, Parks, Water and Environment, Hobart.

Bloom, P.H., & Clark, W.S., (2001). Molt and sequence of plumages of golden eagles and a technique for in-hand ageing. *North American Bird Bander* **26**, 97–116.

Debus, S., (2012). *Birds of prey of Australia*. CSIRO Publishing, Clayton South.

Olsen, P., (2005). *Wedge-Tailed Eagle*. CSIRO Publishing, Collingwood.

Ridpath, M.G., & Brooker, M.G., (1986). Age, movements and the management of the wedge-tailed eagle, *Aquila audax*, in arid Western Australia. *Wildlife Research* **13**, 245–260.


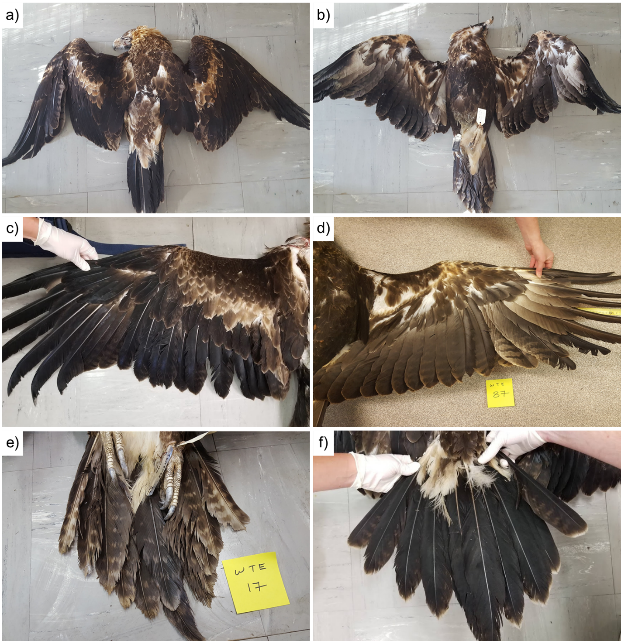


Figure A.1. Example images of Tasmanian wedge-tailed eagles classified as *pre-adult* showing the plumage characteristics; a) dorsal image showing pale brown across back and pale nape, b) ventral image showing pale elements on contour feathers, c) dorsal wing showing wing bar and barring on remiges, d) ventral wing showing barring on remiges, e) ventral tail showing barring on rectrices and pale tail coverts, f) dorsal tail showing pale tail coverts and barring on rectrices.


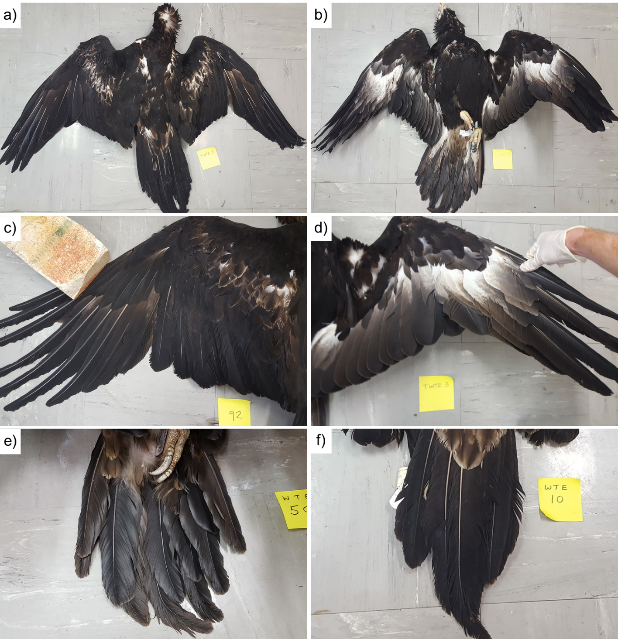


Figure A.2. Example images of Tasmanian wedge-tailed eagles classified as *adult* showing the plumage characteristics; a) dorsal image showing dark contour feathers across back, b) ventral image showing dark contour feathers across body, c) dorsal wing showing narrow wing bar, d) ventral wing showing pale base to flight feathers, e) ventral tail and f) dorsal tail showing brown tail coverts and lack of barring on rectrices.

Appendix B

*Ammunition lead^207/206^ isotope signatures*

Table B. Brands of ammunition available in Tasmania for which there is published information on the respective lead^207/206^ isotope signatures^a^

| Ammunition brand | No. bullets | Country of manufacture | Lead^207/206^ range |
| --- | --- | --- | --- |
| CCI | 48 | USA | 0.8252–0.8351 |
| Federal | 6 | USA | 0.8169–0.8170 |
| Fiocchi | 65 | Italy | 0.8507–0.8638 |
| Lappua | 47 | Finland | 0.8481–0.8640 |
| PMC | 41 | Mexico | 0.8306–0.8901 |
| Remington | 20 | USA | 0.8070–0.8147 |
| RWS | 58 | Germany | 0.8435–0.8885 |
| Sellier & Bellot | 5 | Czech Republic | 0.8582–0.8584 |
| SK | 5 | Germany | 0.8647–0.8649 |
| Winchester | 89 | Australia | 0.9195–0.9561 |
| Winchester | 25 | USA | 0.8079–0.9529 |
|  |  |  |  |

^a^Ammunition isotope information presented is from Sjåstad *et al.* (2014). The number of bullets Sjåstad *et al.* (2014) analyzed from each brand, the corresponding country of manufacture, and the range in lead^207/206^ isotope ratios are presented.

Appendix C

*Correlation between liver and femur lead*


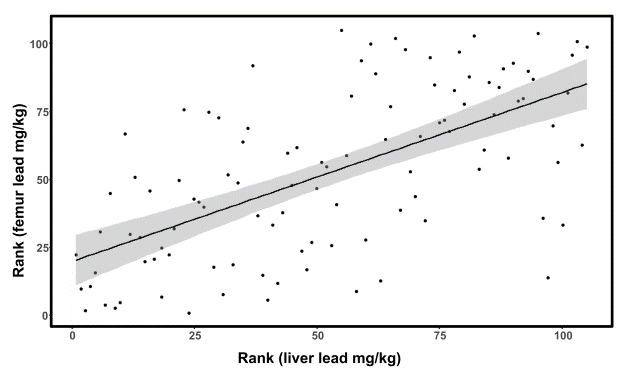


Figure C.1. Association between lead concentrations in liver and femur samples from Tasmanian wedge-tailed eagle carcasses where both sample types were collected (Kendall’s tau = 0.438, *p* < 0.01, z = 6.623, n = 105). The Theil-Sen estimator line is presented with 95% CI indicated by the shaded area.


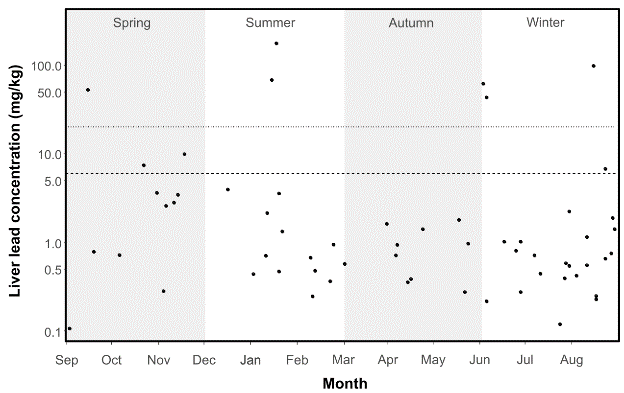
*Seasonal patterns in lead exposure*

Figure C.2. Liver lead concentrations of Tasmanian wedge-tailed eagle carcasses in relation to the day of the year the carcass was retrieved (n = 61). The plot is presented on a log scale for graphical representation of the data. The shade of the plot differentiates the seasons. The horizontal lines indicate lead exposure thresholds (values above the dashed line specify elevated liver lead levels, values above the dotted lines specify severe liver lead levels).


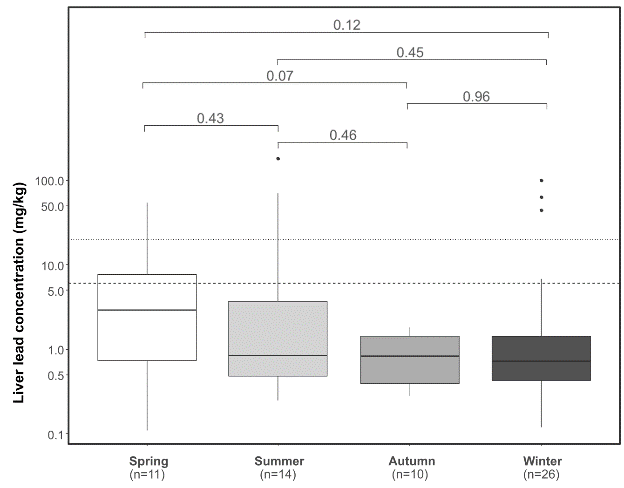


Figure C.3. Boxplot of liver lead concentration for Tasmanian wedge-tailed eagle carcasses retrieved in different seasons. Whiskers are extended to maximum values within 150% of the interquartile range, values beyond this are plotted individually as outliers. The horizontal lines indicate lead exposure thresholds (values above the dashed line specify elevated liver lead levels, values above the dotted lines specify severe liver lead levels). The significance (*p* value) of pairwise comparisons between seasons are shown top.

*Influence of age and season on liver lead concentrations*

We used a multiple linear regression to assess the effect of the age of the eagle and the season the carcass was found on liver lead concentration. This analysis repeated the Wilcoxon rank sum tests in the main manuscript but used a subset of 60 individuals for which age and season data were available. Liver lead concentrations were log10 transformed to improve normality of the data. The residuals of the model were tested for homoscedasticity, normality, linearity, and influential data points. The model was compared with the null model using an ANOVA to assess any influence of these factors on liver lead concentrations.

The multiple linear regression resulted in a non-significant regression equation (*F*_3,56_ = 0.941, *p* = 0.427, *R^2^* = 0.048) and the model was not significantly different to the null model (*p* = 0.589). Neither age nor season were significant predictors of liver lead concentration (see Table C). This is the same result as obtained by the non-parametric Wilcoxon rank sum tests run on the full data set. Model errors were not normally distributed, but other model assumptions were met.

Table C. Coefficients, standard errors, and t-statistics for the linear regression model^a^

| **Parameter** | **Estimate** | **Std. error** | ***t*** | ***Pr*(>\|t\|)** |
| --- | --- | --- | --- | --- |
|  |  |  |  |  |
| Intercept | -0.182 | 0.703 | -0.259 | 0.796 |
| Age: Pre-adult | -0.115 | 0.572 | -0.201 | 0.841 |
| Season: Spring | 1.092 | 0.738 | 1.479 | 0.145 |
| Season: Summer | 0.828 | 0.701 | 1.181 | 0.243 |
| Season: Winter | 0.413 | 0.634 | 0.651 | 0.517 |
|  |  |  |  |  |

^a^Where the dependent variable is log10 transformed liver lead concentration and the independent variables are the age of the eagle and the season in which the eagle was found dead or moribund.

*Isotopic differences between liver exposure categories*


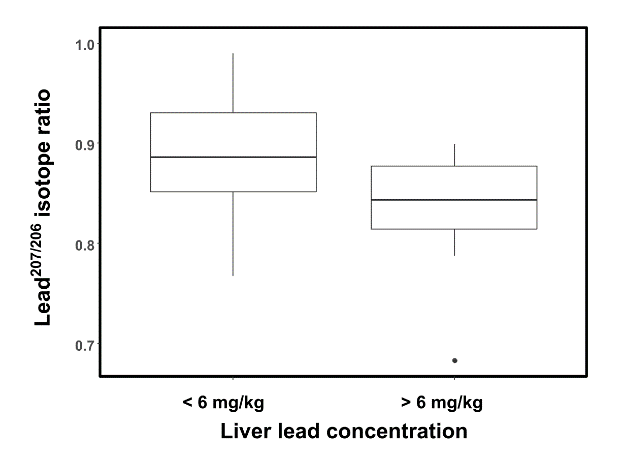


Figure C.4. Box plot of the differences in lead^207/206^ isotope ratios between Tasmanian wedge-tailed eagles with low (< 6 mg/kg, n = 95) and elevated (> 6 mg/kg, n = 11) liver lead levels. Whiskers are extended to maximum values within 150% of the interquartile range, values beyond this are plotted individually as outliers.

*Isotopic patterns in lead detected in nestling blood*


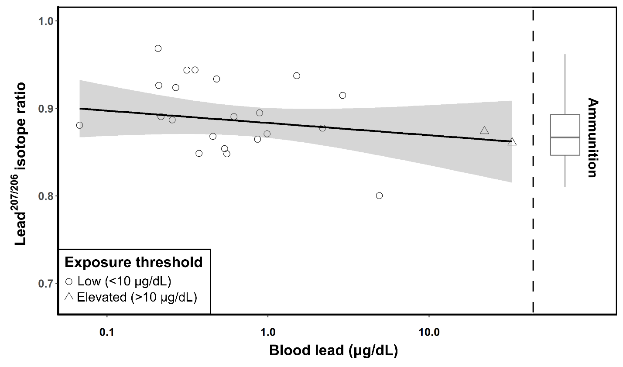


Figure C.5. Relationship between the lead^207/206^ isotope ratio and the concentration of lead in Tasmanian wedge-tailed eagle nestling blood samples with detected lead levels (n = 23). The x axis is presented on the log scale. The Theil-Sen estimator line is presented with 95% CI indicated by the shaded area. The range in published ammunition isotope signatures (Sjåstad *et al.*, 2014) of bullet brands available in Tasmania is shown on the right.
